# NO Binding Energies to and Diffusion Barrier on Pd Obtained with Velocity-Resolved Kinetics

**DOI:** 10.1021/acs.jpcc.1c02965

**Published:** 2021-05-24

**Authors:** Dmitriy Borodin, Igor Rahinov, Jan Fingerhut, Michael Schwarzer, Stefan Hörandl, Georgios Skoulatakis, Dirk Schwarzer, Theofanis N. Kitsopoulos, Alec M. Wodtke

**Affiliations:** † Institute for Physical Chemistry, Georg-August University of Goettingen, Tammannstraße 6, 37077 Goettingen, Germany; ‡ Department of Dynamics at Surfaces, Max Planck Institute for Biophysical Chemistry, Am Fassberg 11, 37077 Goettingen, Germany; § Department of Natural Sciences, The Open University of Israel, 4353701 Raanana, Israel; ∥ Department of Chemistry, University of Crete, Heraklion, Greece; ⊥ Institute of Electronic Structure and Laser − FORTH, Heraklion, Greece; # International Center for Advanced Studies of Energy Conversion, Georg-August University of Goettingen, Tammannstraße 6, 37077 Goettingen, Germany

## Abstract

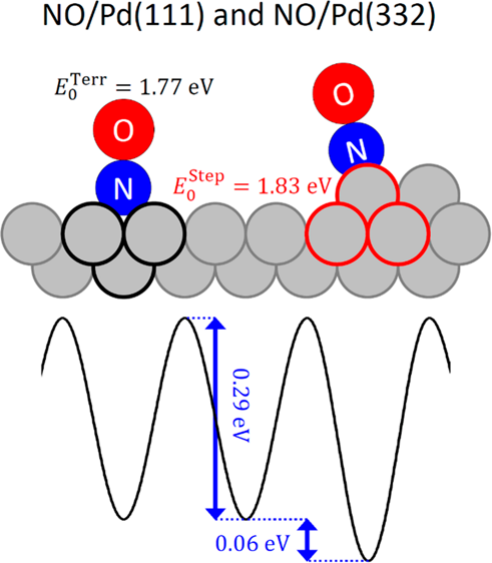

We report nitric oxide (NO) desorption rates from Pd(111) and Pd(332) surfaces measured with velocity-resolved kinetics. The desorption rates at the surface temperatures from 620 to 800 K span more than 3 orders of magnitude, and competing processes, like dissociation, are absent. Applying transition state theory (TST) to model experimental data leads to the NO binding energy *E*_0_ = 1.766 ± 0.024 eV and diffusion barrier *D*_T_ = 0.29 ± 0.11 eV on the (111) terrace and the stabilization energy for (110)-steps Δ*E*_ST_ = 0.060_–0.030_^+0.015^ eV. These parameters provide valuable benchmarks for theory.

## Introduction

Binding energies of molecules at surfaces serve as important descriptors, for screening heterogeneous catalysts. This exploits the well-known correlation of catalytic activity and binding strength realized in the early 20th century by Paul Sabatier.^1,2^ Nowadays, the screening is done using electronic structure calculations based on density functional theory (DFT) with the generalized gradient approximation (GGA).^3−5^ Although DFT-GGA often yields results in agreement with experimental binding energies,^3^ there are examples where it fails. The prediction of the wrong binding site for CO on Pt(111) stands out—“The CO/Pt(111) Puzzle”.^6^ Exchange-correlation functionals at the GGA level predict CO to be bound at the 3-fold hollow site of Pt(111); however, CO binds to the top site. This system has been tackled by various theoreticians, and improvements have been developed,^7−9^ concluding that GGA-related overbinding errors are enhanced at sites with high coordination.

This problem became apparent because CO prefers binding at low coordinated sites. It is likely that GGA calculations of chemisorption energies of NO have similar problems.^10^ However, NO prefers binding at the hollow site of fcc(111) metals in agreement with predictions of DFT-GGA. Hence, GGA errors in the calculated chemisorption energies of NO can only be detected by direct comparison to precise experimental benchmarks. Although NO binding energies can be obtained from calorimitery,^11,12^ competing decomposiiton has prevented any accurate experimental determinations up to now.

The interaction of NO with Pd exemplifies this situation. Here, despite earlier incorrect assignments,^13^ theory and experiment now agree on the preferred binding site;^14^ unfortunately, accurate experimental binding energies are not available. In previous work, NO/Pd(111) binding energies were derived from temperature-programmed desorption (TPD)^15−17^ over a narrow temperature range and under conditions where the rates of NO decomposition and desorption are comparable.^15^ Hence, the reported values (1.5–1.9 eV) are of little use for rigorous benchmarking of theoretical predictions.

In this work, we overcome these experimental problems using velocity-resolved kinetics and are able to use experimental desorption rates to derive the NO chemisorption energy and diffusion barrier on Pd(111) terraces as well as the stabilization energy on steps of Pd(332). Velocity-resolved kinetics is an improvement over previous methods as desorption rates are obtained at higher temperatures and over a broader temperature range. Because of this, desorption rates span over 3 orders of magnitude, and NO decomposition is absent. From the absolute magnitude and the temperature dependence of the desorption rate, we determine the binding energy and the entropy of the adsorbed molecules. Using a model potential energy surface describing diffusion and step stabilization, we obtain an accurate adsorbate partition function as an input to transition state theory (TST). This leads to an accurate binding energy and diffusion barrier. Complementary experiments on a stepped Pd(332) surface clarify an open dispute between experiment and theory. Past experiments suggest that NO is more stably bound at terraces,^17^ in contrast to theoretical predictions.^18^ We find that NO has an energetic preference for steps.

## Methods

Similar to our previously described experiments,^19−21^ an ∼30 μs long pulsed supersonic molecular beam of NO (2–20% NO in He) passed through two differential pumping stages and entered the surface-scattering chamber, at a base pressure of 2 × 10^–10^ mbar, impinging upon the Pd(111) or the Pd(332) surface (MaTeck GmbH) at an incidence angle of 30° from the surface normal. The surface was prepared by sputtering with Ar^+^ (3 keV) for 10 min and subsequent annealing at 1070 K for 15 min. The cleanliness of the sample was verified with Auger electron spectroscopy. The step density of the Pd(111) crystal was determined using atomic force microscopy to be 0.4 ± 0.1%. The desorbing NO was detected, 20 mm from the surface, using nonresonant multiphoton ionization with a Ti:sapphire laser (35 fs, 0.5 W at 1 kHz). A pulsed homogeneous electric field, formed between two parallel flat meshes (repeller and extractor), projected the ions onto a time-gated MCP detector. The mass-to-charge ratio of the ions was fixed by setting the time gate on the MCP with respect to the pulsed-field extraction. The ion image appearing on the phosphor screen at the back of the MCP detector was recorded with a CCD camera.

The position of each pixel in the image relative to the intersection of the probe laser and surface normal directions corresponds to an NO velocity vector. The velocity information is used to calculate the flight time of the NO from the surface to the ionizing laser spot to more accurately determine the residence time and to convert NO density to flux. The ion images also record thermal background, which was subtracted from the signal using knowledge of the background’s velocity distribution. The flux images at each beam-laser delay are integrated for velocities between 400 and 800 m/s at angles close to the surface normal, suppressing directly scattered NO’s contribution to the kinetic traces. The translational energy distribution of the desorbing molecules was determined by summing all ion images from each beam-laser delay.

## Results

**Figure 1:**
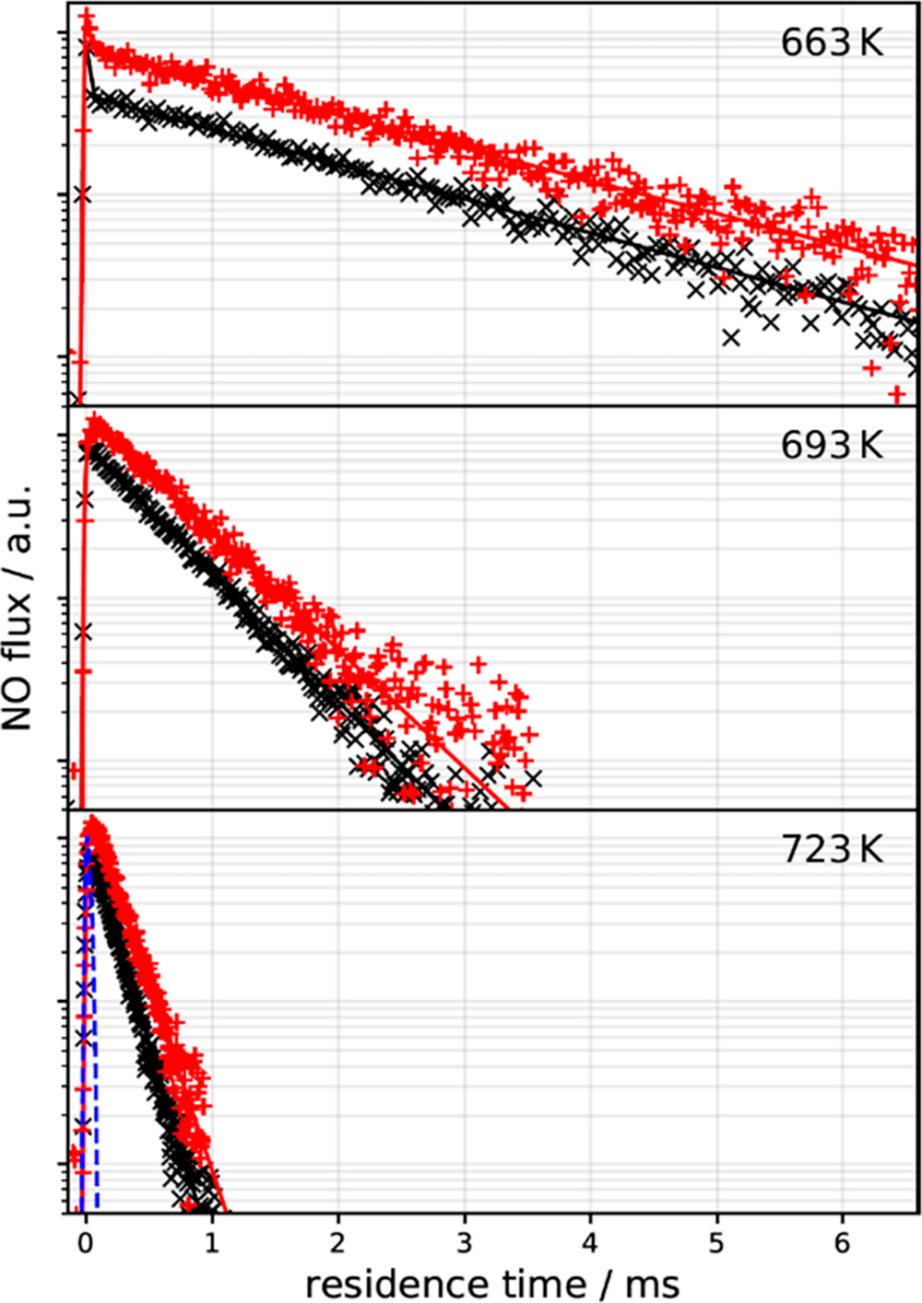
Kinetic traces of NO desorbing from Pd(111) (black ×, experimental data; black −, fits to eq 1) and from Pd(332) (red +, experimental data; red −, fits to eq 1) at various surface temperatures. The dashed blue line denotes the molecular beam dosing function, which determines the temporal resolution of the experiment. The kinetic traces are set apart from one another for clarity.

Figure 1 shows representative kinetic traces for NO desorbing from Pd(111) and Pd(332). The exponential decay is characteristic of a first-order process and is seen over the entire temperature range of this work. NO doses above ≈1 × 10^–2^ ML/pulse and below ≈1 × 10^–3^ ML/pulse are indistinguishable. As the step density of the crystal is 4 ± 1 × 10^–3^ ML, this indicates the absence of step saturation effects in the (111) experiments, seen previously for CO and NO on stepped Pt surfaces.^22,23^ For experiments on the Pd(332) surface, the NO dose from each molecular beam pulse is always below the step density (0.17 ML). The absence of a biexponential kinetic trace at high NO doses indicates that steps do not play a significant role for NO desorption from Pd(111). We do not observe NO decomposition or reaction. In contrast to previous work,^15,17^ the experimental conditions that can be reached with velocity-resolved kinetics allow us to exclusively probe the elementary process of desorption.

To determine the desorption rate constants from the kinetic trace, we fit the flux *f*(*t*) vs residence time *t* using a function representing two contributions
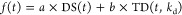
1comprising direct scattering (DS) and a trapping desorption (TD). The DS contribution has the temporal shape of the incident molecular beam, while the TD contribution is a first-order decay convoluted with the molecular beam temporal profile. The fit yields three independent fit parameters: *a*, *b*, the amplitudes of DS and TD, and *k*_d_, the desorption rate constant.

**Figure 2:**
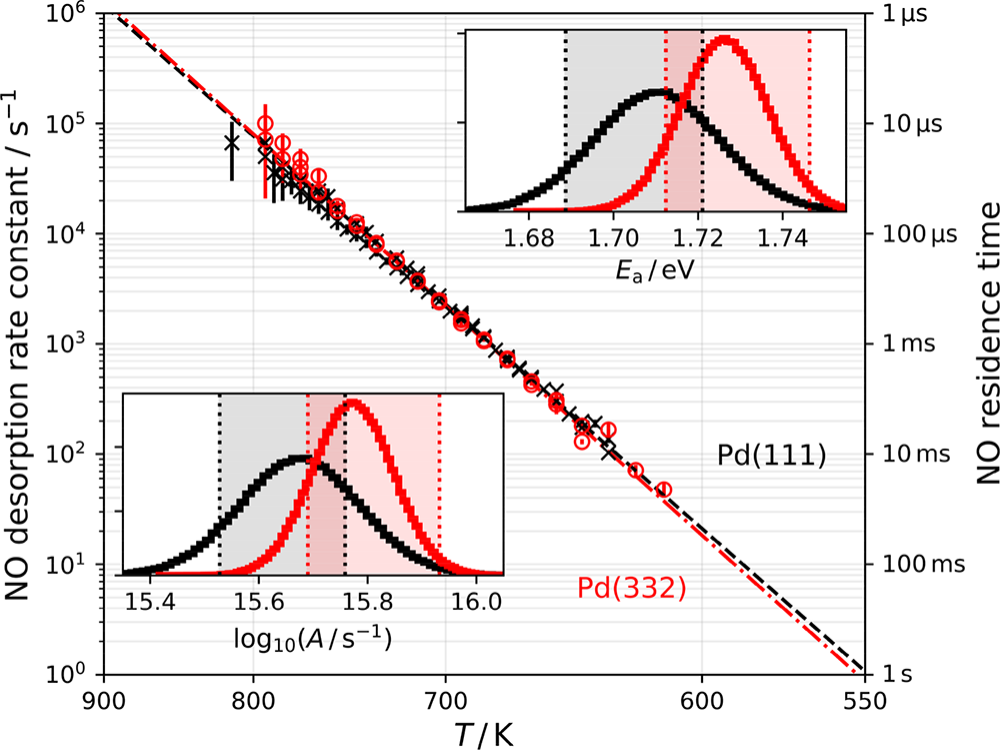
Arrhenius plot of NO desorption rate constants from Pd(111) (black ×, experiment; black ---, Arrhenius error-weighted fit) and Pd(332) (red ○, experiment: red -·-, Arrhenius error-weighted fit). The error bars indicate 95% confidence intervals. The insets show the error distributions (black, Pd(111); red, Pd(332)) for the activation energy, *E*_a_ (upper right), and the prefactor, *A* (lower left). The red and black shaded regions in the insets are the Arrhenius rate parameter predictions from the TST model obtained between 620 and 800 K (see Section 4).

Desorption rate constants were derived for ∼50 surface temperatures between 620 and 800 K for both surfaces (see Figure 2). By fitting the Arrhenius equation, we find that the prefactor and the activation energy, *A* and *E*_a_, differ slightly for experiments done with Pd(111) (*E*_a_ = 1.71 ± 0.03 eV, *A* = 10^15.65±0.20^ s^–1^) versus Pd(332) (*E*_a_ = 1.73 ± 0.02 eV, *A* = 10^15.80±0.15^ s^–1^). Using the covariance matrix of the least-squares fit, we map out the error distribution for *A* and *E*_a_, shown as red and black histograms in the insets in Figure 2.

The velocity-resolved kinetics experiment provides both the desorption rate—yielding the barrier for desorption, and, the translational energy distribution - allowing one to determine the magnitude of the barrier for adsorption. In this case the adsorption has no barrier, and therefore the barrier for desorption is the same as the NO binding energy.

**Figure 3:**
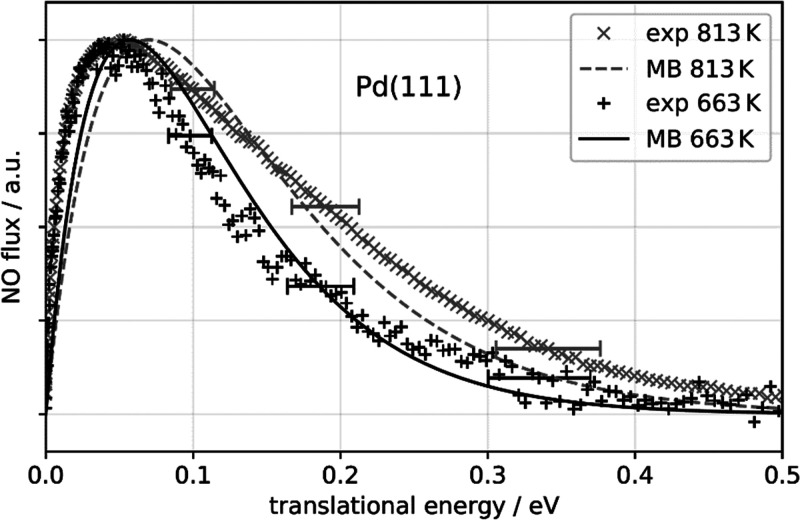
Translational energy distributions of NO molecules desorbing from Pd(111) at surface temperatures of 813 K (×, experiment; ---, 3D Maxwell–Boltzmann distribution at 813 K) and 663 K (+, experiment; −, 3D Maxwell–Boltzmann distribution at 663 K). The experimentally obtained translational energy distributions exhibit effective temperature close (within 10%) to the 3D Maxwell–Boltzmann distributions at the surface temperature. The horizontal error bars indicate the 2σ error of the kinetic energy determination.

Figure 3 shows translational energy distributions of NO desorbing from Pd(111) at two representative temperatures, as well as the thermal 3D Maxwell–Boltzmann distributions. The experimentally observed translational energy distributions are not hyperthermal, indicating the absence of an adsorption barrier. They are similar (within the error bars of translational energy determination) to the thermal Maxwell–Boltzmann distributions, characteristic for a chemisorbed system with high (close to unity) sticking coefficient, weakly dependent on the incident kinetic energy. This conclusion is supported by previous King and Wells measurements of the NO sticking coefficient at Pd(111) using effusive molecular beams.^15^ We observe similar translational energy distributions for Pd(332) experiments (not shown).

## Discussion

The desorption kinetics of NO from Pd single-crystal surfaces has been studied previously with temperature-programmed desorption (TPD); however, the reported rate parameters vary widely, with activation energies between 1.5 and 1.9 eV and prefactors between 10^14^ and 10^18^ s^–1^.^15−17^ It is worth noting that despite various TPD studies arriving at different Arrhenius parameters all reported a TPD spectral peak near ∼520 K for low initial coverages. This suggests that the errors in the reported values of *A* and *E*_a_ are correlated, a typical problem in TPD experiments which probe a narrow temperature range. Another problem with these TPD studies is decomposition of NO—measured rates do not reflect a single elementary kinetic process. Only in the work of Schmick and Wassmuth^15^ has this been explicitly taken into account in the analysis of the TPD rates. The authors found that at the low temperatures of the TPD studies NO decomposes at steps of Pd(111) crystals with an efficiency of ≈50% in the low coverage limit. They estimated the rate parameters for the decomposition process—reporting a prefactor of 4 × 10^11^ s^–1^ and estimating the activation energy for decomposition to be ≈80% of the desorption energy.^15^ In our experiments, we find no evidence of NO decomposition on Pd(111) and Pd(332). Specifically, we observe no signals from N_2_, N_2_O, NO_2_, or O_2_, reported in earlier works.^15,17,24^ We also see no variation in the NO desorption rate even after long NO exposure times. Chemical change of the surface due to decomposition is absent.

This is because the fraction of NO-forming decomposition products φ_dis_(*T*) is strongly temperature dependent. Consider
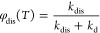
2where *k*_dis_ is the rate constant for dissociation and *k*_d_ is the rate constant for desorption. Using  from our experiment and , following the suggestions of Schmick and Wassmuth,^15^ we find that at the temperatures of the TPD work φ_dis_ (400–500 K) is between 65 and 20%, whereas at the temperatures of our work, φ_dis_ (620–800 K) is 5–1%. An independent upper limit to the decomposition of NO (1%) in our experiments is found from an estimate of N_2_ detection sensitivity. This upper limit is consistent with the uncertainty range of the previous work.^15^

The high quality of the NO desorption rate data made possible by our velocity-resolved methods warrants application of TST to obtain fundamental insights into the experimental observations. Thermal reaction rates are routinely obtained from TST, which places a dividing plane along the reaction coordinate and takes the equilibrium one-way flux through it as the reaction rate. Typically, TST gives an upper limit to the rate constant, *k*_TST_(*T*), as recrossing the dividing plane is neglected. For desorption, it is convenient to place the dividing plane at a large separation from the surface. In that case, the recrossing corrected thermal desorption rate constant is
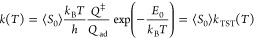
3where *Q*^⧧^ and *Q*_ad_ are the partition functions of the desorbed gas-phase molecule and the adsorbate, respectively. *E*_0_ is then the desorption barrier—in this case, the binding energy—and the thermal sticking coefficient ⟨*S*_0_⟩ provides the recrossing correction.^25^ From the experimental velocity distributions, we surmise that the thermal sticking coefficient is close to unity, consistent with previous experiments.^15^ Thus, we apply ⟨*S*_0_⟩ = 1 for the analysis of desorption rates from both surfaces, such that TST accurately represents the thermal rate.

The partition function of the desorbed gas-phase molecule is then given by
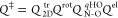
4Here, *Q*_2D_^tr^ is the translational partition function of a 2D ideal gas
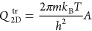
5with *m* being the mass of NO. *A* is the area of the reference cell in which the partition function is defined. It is convenient to think of it as the area of the unit cell of the Pd surface. However, the numerical value of this area cancels out in the calculation of the TST rate constant as it enters both *Q*^⧧^ and *Q*_ad_ in eq 3. Note that translation normal to the surface is associated with the reaction coordinate in TST and does not appear in *Q*^⧧^. *Q*^rot^ is the classical partition function of a rigid rotor
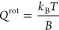
6with *B* being the rotational constant. *Q*_N–O_^*q*HO^ is the vibrational partition function approximated by a quantum harmonic oscillator
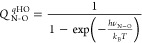
7with ν_N–O_ being the vibrational frequency of the free molecule. *Q*^el^ is the electronic partition function
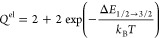
8where Δ*E*_1/2→3/2_ is the energetic separation between the two spin–orbit components of the ground electronic state. Higher electronic states of NO are not populated at the temperature range of this work.

While the partition function for a diatomic in the gas phase is rather straightforward, a more careful approach is required for the adsorbate. Ignoring the surface atom motion, the number of degrees of freedom associated with the adsorbed molecule remains the same as for the gas phase. However, what was previously a free translation along *x*, *y*, and *z* (*x* and *y* run parallel, while *z* runs normal to the surface) becomes a hindered translation or vibration due to interaction with the surface. The situation is similar for degrees of freedom corresponding to gas-phase rotation. NO binds on Pd with its bond perpendicular to the surface,^10^ so rotation around the NO bond axis is unimportant. The N–O stretch frequency of the molecule also changes due to interaction with the metal. While the degrees of freedom may depend weakly on binding site, we make an approximation by neglecting this dependence.

We approximate the hindered translation of NO perpendicular to the surface, the N–O stretch vibration, and the doubly degenerate hindered rotations (in *xz*- and *yz*-planes) as harmonic oscillators. Approximating hindered rotation as vibration is reasonable for chemisorbed molecules like NO and CO, which have rather high rotational isomerization barriers in their most stable configuration.^26^ For the electronic partition function of the adsorbed NO, we use *Q*_ad_^el^ = 2 to account for the spin states. The reduction of the electronic partition function for the adsorbate by nearly a factor of 2, compared to the gas phase, is due to the splitting of the doubly degenerate 2π* orbital of NO into a bonding and an antibonding orbital that results from interactions with the metal orbitals.^10,27^ The high energy antibonding orbital remains unpopulated at the temperatures of our work.

**Figure 4:**
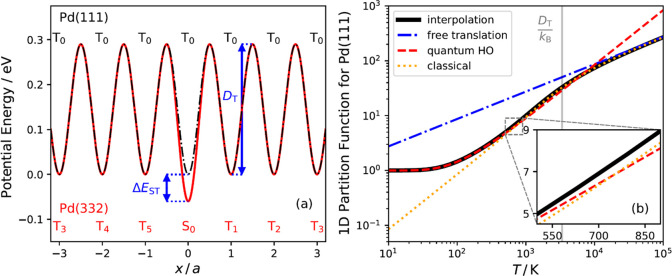
(a) Model potential used to describe the in-plane partition function of NO on Pd(111) (black dash-dotted line) and Pd(332) (red line). *T*_i_ denotes terrace sites, and S_*i*_ denotes step sites. The periodic unit of the potential is defined between two binding sites with the same index. The diffusion barrier between two terrace sites (*D*_T_) and the step-terrace energy difference (Δ*E*_ST_) are indicated in the plot. (b) Partition functions for NO motion parallel to a Pd(111) surface. The most accurate partition function (black line) extrapolates at low temperature to the quantum harmonic oscillator (red dashed line), at intermediate temperatures to the prediction of eq 9 (orange dotted line), and at high temperatures to the one-dimensional free translational partition function (blue dashed line). The inset shows a zoom-in of the temperature range in our analysis.

The proper description of NO motion parallel to the surface depends on temperature. It can be described at low temperature by vibration using the harmonic oscillator partition function or at high temperature as a hindered translation by the classical partition function given by
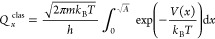
9where *V*(*x*) is the molecule–surface interaction potential. In eq 9, the integration is done for the periodic unit of the surface. For the purpose of demonstration in Figure 4b, we use √*A* = 2.77 Å to calculate the partition functions. Note that the harmonic oscillator will fail to describe the density of states properly when the diffusion barrier between two binding sites is low compared to the thermal energy (see the red dashed line in Figure 4b). Likewise, the classical partition function will predict unphysically low values at low temperatures (see the orange dotted line in Figure 4b).

To accurately cover all temperatures, we first construct a periodic potential for both surfaces. The one-dimensional potential for Pd(111) is modeled by
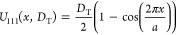
10where the minima that represent the NO binding sites on the surface repeat, with each *a* = 2.77 Å being the interatomic distance of Pd atoms on the surface. The amplitude of this function, *D*_T_, is the diffusion barrier between two binding sites. The Pd(111) potential is shown as a black dashed-dotted line in Figure 4a. The NO/Pd(111) potential along the *y*-axis is assumed to be identical to that along the *x*-axis.

To construct the potential for Pd(332), we define the *x*-axis as the coordinate perpendicular to the steps of Pd(332). The one-dimensional Pd(332) potential along this axis is given by
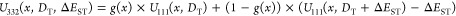
11Here Δ*E*_ST_ is the energy difference between the step and terrace site, and *g*(*x*) is a cutoff function that is used to smoothly stitch the two basis functions at the step. A Pd(332) one-dimensional potential, generated in this way, is shown in Figure 4a as the red solid line. The NO/Pd(332) potential along the *y*-axis (parallel to the steps) is identical to the NO/Pd(111) potential.

Therefore, we define the in-plane (two-dimensional) potential for Pd(111) as
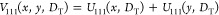
12and for Pd(332) as
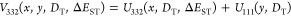
13We note that the potential functions defined in this way do not reproduce the symmetry of the Pd(111) surface; however, we expect this simplification has no effect on the calculated entropy.^28^ With this potential expression, the in-plane partition function, *Q*_*xy*_, becomes separable and is given by the product of two one-dimensional partition functions *Q*_*x*_ and *Q*_*y*_, associated with the corresponding potential energy contribution.

The construction of an accurate one-dimensional partition function (black line of Figure 4b), *Q*_*x*_ (or equivalently *Q*_*y*_), is done following the procedure originally suggested for describing the heat capacity contribution of the hindered rotation of ethane in the gas phase.^29,30^ This approach has been suggested earlier for applications of surface reactions,^28^ and it is considered an accurate method to describe the partition function.^31^ We define *Q*_*x*_ as
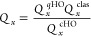
14where *Q*_*x*_^cHO^ is the partition function of the classical harmonic oscillator for hindered translation (eq 9). As mentioned above, *Q*_*x*_^qHO^ is a poor approximation of *Q*_*x*_ at high temperatures, while *Q*_*x*_^clas^ is not an appropriate approach at low temperatures. Dividing the product of *Q*_*x*_^*q*HO^ and *Q*_*x*_^clas^ by the *Q*_*x*_^cHO^ is used as a trick to circumvent the inaccuracies associated with the partition function at different temperature limits. At high temperatures, the quantum harmonic oscillator partition function becomes its classical counterpart, while at low temperatures the classical partition function becomes equal to the classical oscillator partition function. Note that the hindered translation frequencies, ν_*x*_, used in *Q*_*x*_^*q*HO^ and *Q*_*x*_^cHO^ are determined from *V*(*x*) used to calculate *Q*_*x*_^clas^—this is required for self-consistency of the interpolation function *Q*_*x*_. Using this approach, it is only the diffusion barrier that determines the magnitude of *Q*_*x*_. In Figure 4b, we show that *Q*_*x*_ (black bold line) is converging to the correct limiting cases at low (*Q*_*x*_^*q*HO^, red-dashed line) and high temperatures (*Q*_*x*_^clas^, orange-dotted line). The total partition function for the adsorbed NO is given by
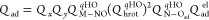
15All the parameters needed to evaluate the partition function of NO_(g)_ and NO_ad_ are summarized in Table 1. We note in passing that the partition functions presented here are valid only in the zero-coverage limit relevant to our experiments. Detailed discussion of partition functions and the derived thermodynamic state functions capturing the effects of surface coverage can be found in refs (28 and 32).

**Table 1:** Parameters Required to Evaluate the Partition Functions for NO_(g)_ and NO_ad_ at Pd(111) and Pd(332)^a^

property	NO_(g)_	NO/Pd(111)	NO/Pd(332)	comment
*ν*_N–O_/cm^–1^	1904^33^	1540^13^	1540*	*value assumed to be the same as for terrace, see text
*B*/cm^–1^	1.67^33^			
*Q*_el_	see eq 8	2	2	
Δ*E*_1/2**→**3/2_/cm^–1^	120^34^			
*ν*_M–NO_/cm^–1^		330^13^	(ν*_x_*_,step_*/ν_*x*,terr_* ) × 330	*values are obtained from the fit to Pd(111) and Pd(332)
*ν*_hrot_/cm^–1^		380*	(ν*_x_*_,step_*/ν_*x*,terr_*) × 380	*value for hindered rotational frequency from NO/Pt(100)^35^
⟨*S*_0_⟩		1	1	
*a*/Å		2.77	2.77	

a The frequencies of the out-of-plane hindered translation (ν_M–NO_) and the hindered rotation frequency (ν_hrot_) upon adsorption to steps are not reported. We assume that they scale proportionally to the in-plane hindered translation vibrational frequency (ν_*x*_). The scaling on Pd(332) is only done if steps are the most stable binding site, which is not assumed a priori in the fitting procedure.

**Figure 5:**
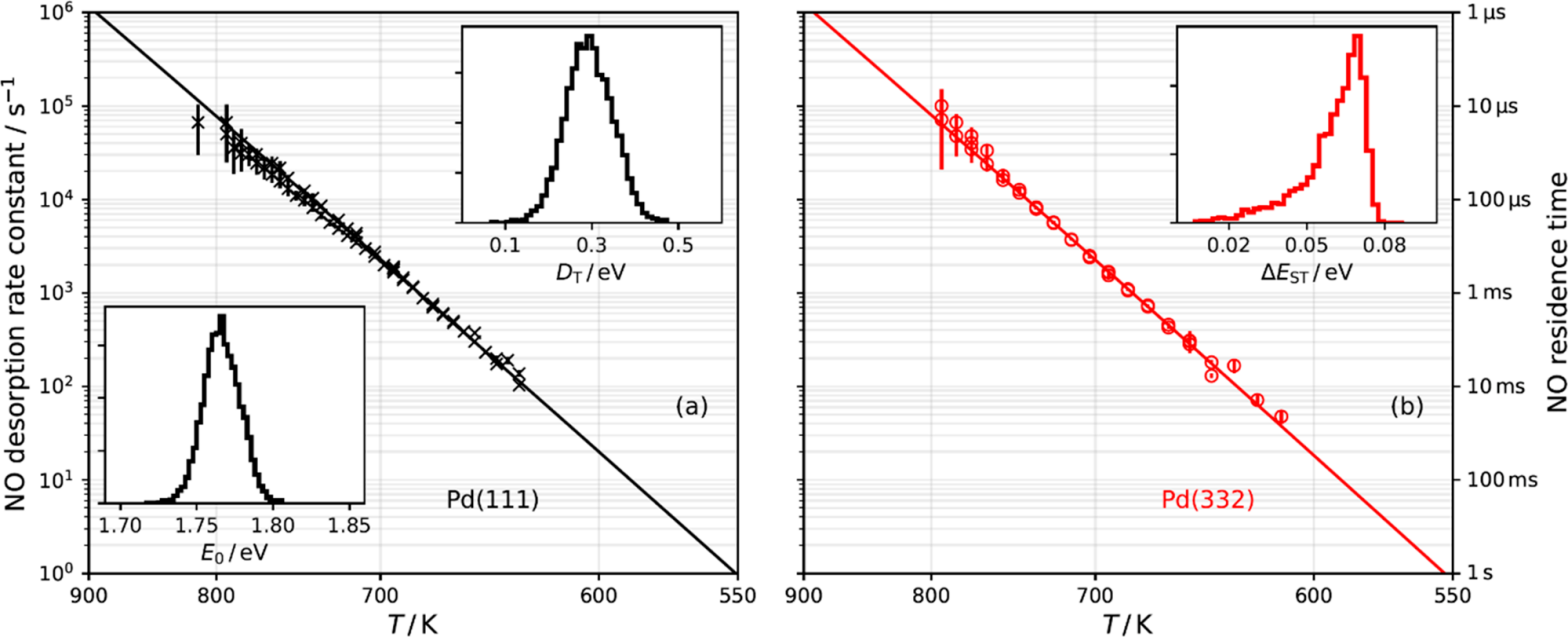
(a) Desorption rate constants from Pd(111) (black ×) are shown with the corresponding TST model fit (black −). The parameter distributions obtained from the fit to the data, NO binding energy *E*_0_, and NO diffusion barrier *D*_T_ are shown in the insets. (b) Desorption rate constants from Pd(332) (red ○) are shown with the corresponding TST model fit (red −). The obtained distribution of the NO step-terrace energy difference at Pd(332) is shown in the inset.

Next we use these partition functions to construct the TST rate expression, with which we fit the measured NO desorption rates. For desorption rates from Pd(111), we fit using only two parameters, the diffusion barrier, *D*_T_, and the binding energy on the terrace, *E*_0_. The best fit of the TST model (black line) to the measured rate constants (black crosses) is shown in Figure 5a. To evaluate uncertainty, the parameter distribution of *D*_T_ and *E*_0_ is sampled by random numbers from a multivariate Gaussian distribution using the covariance matrix that was obtained from the least-squares fit. The parameter distributions are shown as insets in Figure 5a. This analysis leads to our final results, *D*_T_ = 0.29 ± 0.11 eV and *E*_0_ = 1.766 ± 0.024 eV, where the error bars represent 95% confidence intervals.

For our analysis, we use the hindered rotational frequency of NO that had been observed on Pt(100) earlier.^35^ It is known that CO on transition metal surfaces has hindered rotational frequencies that are similar within ∼40 cm^–1^.^36−38^ We expect the same to be valid for NO. We have investigated the introduced error by modifying the hindered rotational frequency by ±40 cm^–1^ for the fit and find that *D*_T_ and *E*_0_ remain within the reported error range.

We also analyzed the Pd(332) rate data with the TST model. We used the correlated distribution of *D*_T_ and *E*_0_ obtained from the least-squares fitting of the Pd(111) rate data to determine the distribution of step-terrace stabilization energies Δ*E*_ST_ from a fit to Pd(332) rate data. The results are shown in the inset of Figure 5b. The best fit to the Pd(332) rate data is shown in Figure 5b as a red solid line. The results of this analysis show that NO is energetically stabilized at steps of Pd(332) by Δ*E*_ST_ = 0.060_–0.030_^+0.015^ eV. Table 2 summarizes all of the results of the TST-based desorption rate analysis.

**Table 2:** Rate and Energy Parameters Determined in This Work for the NO/Pd(111) and the NO/Pd(332) Systems^a^

property	Pd(111)	Pd(332)	comment
*A*/s^–1^	10^15.65±0.20^	10^15.80±0.15^	Arrhenius fit
*E*_a,d_/eV	1.71 ± 0.03	1.73 ± 0.02	Arrhenius fit
*E*_0_/eV	1.766 ± 0.024	1.766 ± 0.024	binding energy at the terrace, TST fit to Pd(111) data
*D*_T_/eV	0.29 ± 0.11	0.29 ± 0.11	TST fit to Pd(111) data
Δ*E*_ST_/eV	**–**	0.060_–0.030_^+0.015^	TST fit to Pd(332) data
*ν*_*x*_/cm^–1^	82 ± 16	90 ± 18	hindered translational frequency from *D*_T_ and Δ*E*_ST_
*ν*_*y*_/cm^**–1**^	82 ± 16	82 ± 16	hindered translational frequency from *D*_T_

a The hindered translational frequencies (ν_*x*_ and ν_*y*_) result from the one-dimensional potential and the fitted diffusion barrier. The uncertainties indicate a 95% confidence interval.

It is worth noting that that the difference in the activation energies (∼0.02 eV) obtained from Arrhenius fits to the NO desorption rates on Pd(111) and Pd(332) in Figure 2 might lead one to believe that that the energy preference for steps is negligible. However, this naïve conclusion neglects entropy considerations. Our modeling approach, self-consistently, links the energetic difference between two binding sites with the associated change in the density of states. Thus, energetic stabilization of NO at the step leads to a decrease in the local density of states, compared to a terrace site. As a consequence, Δ*E*_ST_ is substantially larger than the difference of activation energies, as the entropic contribution to the desorption rate also differs between terraces and steps. We emphasize that the absolute upper limit of NO step preference on Pd(332) is 0.08 eV—higher energy values cannot be compensated by entropic arguments.

For quantitative comparison, we have used our TST predicted rates to obtain Arrhenius activation energies and prefactors using
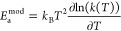
16
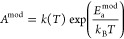
17We obtain *E*_a_^mod^ and *A*^mod^ for Pd(111) and Pd(332) between 800 and 620 K and show the predicted values as gray and red shaded regions in the insets of Figure 2. Our results are consistent with the small difference of activation energies and prefactors obtained from the Arrhenius fits, indicating the high fidelity of the TST analysis.

## Conclusions

In this work, we have reported experimental desorption rates of nitric oxide from Pd(111) and Pd(332) between 620 and 800 K. We employed molecular-beam surface scattering with velocity-resolved kinetics, which allowed us to work at conditions where NO decomposition is substantially suppressed compared to desorption. We have applied TST analysis to the accurate desorption rate data to determine the NO binding energy to Pd(111) (1.766 ± 0.024 eV), its diffusion barrier on (111) terraces (0.29 ± 0.11 eV), and the NO stabilization energy at steps of Pd(332) (0.060_–0.030_^+0.015^ eV). While it is well-known that the prediction of binding site preference for CO at transition metal surfaces can only be achieved with DFT methods beyond the GGA level,^6−10,23^ little effort has been made to investigate similar effects on chemisorption energies for NO. DFT functionals at the GGA level predict a broad range of binding energies: 2.07–2.34 eV (PW91),^39−41^ 2.21 eV (PBE),^10^ and 1.49–1.84 eV (RPBE).^39−41^ The work of Huang and Mason^10^ suggests, that by using the DFT+*U* method, the GGA chemisorption energies of NO at Pd(111) are corrected by 10–15%.^10^ With this correction, they suggest a binding energy of 1.95 eV (initially using the PBE functional, 2.21 eV) which is still 0.16 eV higher than the upper boundary of the binding energy derived from our measurements.

These differences likely underestimate the error in the DFT-GGA binding energies. The NO binding energy determined in this work applies to the low NO coverage limit, whereas most DFT-GGA calculations were conducted for 0.25 ML coverage, where NO–NO repulsion energies are important.^15,40^ Hence, the calculations presented above will yield somewhat higher values of the binding energy in the limit of low NO coverage, making the agreement between theory and experiment even less satisfying.

We have also been able to estimate the NO diffusion barrier on Pd(111) to be 0.29 ± 0.11 eV. To the best of our knowledge, this is the first experimental report of this parameter. DFT calculations report values of 0.22 eV (PBE with DFT+*U*),^10^ 0.32 eV (PBE and RPBE),^40,41^ and 0.36 eV (PW91).^41^ All are in good agreement with our results. While binding energies are very sensitive to the choice of the functional, diffusion barriers show little dependence, indicating that the GGA overbinding clearly demonstrated by the comparison to our experimental binding energy is approximately independent of the position along the diffusion pathway.

We have determined the step-terrace energy preference for NO at Pd(332) to be 0.060_–0.030_^+0.015^ eV. Despite this energy preference, the desorption rates on Pd(111) and Pd(332) are very similar. The reason for this is that with the energetic stabilization of NO at steps also the local density of states is consequently reduced. These two effects cancel mutually and lead to similar magnitude of the desorption rates with weak differences in their temperature dependence. Small energetic preference for steps, such as in the NO/Pd(332) system, tends thus to be missed in the analysis of experimental desorption data.

The energetic stabilization on steps is contrasted by previous experimental work that concluded stronger binding of NO at terraces.^17^ However, we note that those experiments rely on an assumed assignment of HREELS vibrational spectra, and furthermore, the step type associated with the (211) crystal used in that work was different than the step type found on (332) crystals. So far, there have been no DFT calculations that can be directly compared to our work. However, DFT calculations on similar step sites find that NO binding is stabilized by 0.06 eV compared to the terrace site,^39^ quite close in magnitude to our reported value of Δ*E*_ST_.
